# Dermatitis during Spaceflight Associated with HSV-1 Reactivation

**DOI:** 10.3390/v14040789

**Published:** 2022-04-11

**Authors:** Satish K. Mehta, Moriah L. Szpara, Bridgette V. Rooney, Douglass M. Diak, Mackenzie M. Shipley, Daniel W. Renner, Stephanie S. Krieger, Mayra A. Nelman-Gonzalez, Sara R. Zwart, Scott M. Smith, Brian E. Crucian

**Affiliations:** 1 JES Tech, Human Health and Performance Directorate, Houston, TX 77058, USA; 2 Center for Infectious Disease Dynamics, Departments of Biology, Biochemistry and Molecular Biology, Huck Institute for the Life Sciences, Pennsylvania State University, University Park, PA 16802, USA; moriah@psu.edu (M.L.S.); shipleym8@gmail.com (M.M.S.); dwr19@psu.edu (D.W.R.); 3 GeoControl Systems, Human Health and Performance Directorate, Houston, TX 77054, USA; breedge23@yahoo.com; 4 Aegis Aerospace, Human Health and Performance Directorate, Houston, TX 77058, USA; douglass.m.diak@nasa.gov; 5 KBR, Human Health and Performance Directorate, Houston, TX 77058, USA; stephanie.s.krieger@nasa.gov (S.S.K.); mayra.a.nelman@nasa.gov (M.A.N.-G.); 6 University of Texas Medical Branch, Preventive Medicine and Population Health, Galveston, TX 77555, USA; sara.zwart-1@nasa.gov; 7 National Aeronautics and Space Administration (NASA) Johnson Space Center, Human Health and Performance Directorate, Houston, TX 77058, USA; scott.m.smith@nasa.gov (S.M.S.); brian.crucian-1@nasa.gov (B.E.C.)

**Keywords:** herpes, viral reactivation, spaceflight, dermatitis, stress, immune depression

## Abstract

Human alpha herpesviruses herpes simplex virus (HSV-1) and varicella zoster virus (VZV) establish latency in various cranial nerve ganglia and often reactivate in response to stress-associated immune system dysregulation. Reactivation of Epstein Barr virus (EBV), VZV, HSV-1, and cytomegalovirus (CMV) is typically asymptomatic during spaceflight, though live/infectious virus has been recovered and the shedding rate increases with mission duration. The risk of clinical disease, therefore, may increase for astronauts assigned to extended missions (>180 days). Here, we report, for the first time, a case of HSV-1 skin rash (dermatitis) occurring during long-duration spaceflight. The astronaut reported persistent dermatitis during flight, which was treated onboard with oral antihistamines and topical/oral steroids. No HSV-1 DNA was detected in 6-month pre-mission saliva samples, but on flight day 82, a saliva and rash swab both yielded 4.8 copies/ng DNA and 5.3 × 10^4^ copies/ng DNA, respectively. Post-mission saliva samples continued to have a high infectious HSV-1 load (1.67 × 10^7^ copies/ng DNA). HSV-1 from both rash and saliva samples had 99.9% genotype homology. Additional physiological monitoring, including stress biomarkers (cortisol, dehydroepiandrosterone (DHEA), and salivary amylase), immune markers (adaptive regulatory and inflammatory plasma cytokines), and biochemical profile markers, including vitamin/mineral status and bone metabolism, are also presented for this case. These data highlight an atypical presentation of HSV-1 during spaceflight and underscore the importance of viral screening during clinical evaluations of in-flight dermatitis to determine viral etiology and guide treatment.

## 1. Introduction

Over the last two decades, our studies have shown that astronauts exhibit persistent immune system dysregulation due to stress and other unique features associated with spaceflight [1,2,3]. Further, we have illustrated that multiple herpesviruses persistently reactivate in astronauts during space missions. This is evidenced by the shedding of viral DNA in body fluids, namely saliva, before, during, and after both short (up to 16 days) and long (≥180 days) duration space missions [4,5]. About 50% of astronauts reactivate and shed viral DNA for one or more of the nine known human herpesviruses during and after spaceflight [6]. Four common herpes viruses that have been detected during space flight include Epstein Barr virus (EBV), Varicella Zoster virus (VZV), herpes simplex virus 1 (HSV-1), and cytomegalovirus (CMV). Though the typical shedding of viral DNA is asymptomatic in most astronauts regardless of mission duration, live/infectious virus has been recovered in some cases. Whether crew develop symptoms or not, virus reactivation rates increase with spaceflight duration. This represents a significant health risk to crew assigned to extended missions to Mars and beyond.

Stress depresses immunity, which contributes to latent herpesvirus reactivation. This correlation has been demonstrated in astronauts during space missions of variable durations over the last 20 years. Activation of the hypothalamic-pituitary-adrenal (HPA) axis and the sympathetic-adrenal-medullary (SAM) axis during spaceflight results in increased levels of stress hormones, including cortisol, dehydroepiandrosterone (DHEA), epinephrine, and norepinephrine [7,8,9]. Increases in these stress hormones, along with decreased cell-mediated immunity (as evidenced by decrements in cytotoxic T-cell function [10] and Th1 > Th2 cytokine shifts [11]), are contributing factors in the reactivation of latent herpes viruses in astronauts [12].

Here, we report for the first-time detection of HSV-1 in saliva and in skin lesion samples. These samples were taken from an astronaut suffering persistent dermatitis lasting approximately 200 days during a long-duration spaceflight (≥180 days) aboard the International Space Station (ISS). This astronaut reported rash onset at/around flight day 24 (FD24), with red, bumpy lesions appearing on the hands, arms, chest, back, and neck. The astronaut started antihistamines at that time, along with topical and oral hydrocortisone (fluocinonide 0.05% applied twice daily and fexofenadine HCl 180 mg daily). Twenty-nine days later, the astronaut developed a cold sore at FD53 that was subsequently treated with Valtrex (2000 mg PO q12h for 2 days). The astronaut continued to have rash along hands, arms, chest, back, and neck from which a lesion swab sample was collected at FD82. Finally, the astronaut was treated again for a cold sore with Valtrex on FD168. Interestingly, this astronaut had a previously documented history of atopic dermatitis [13]. While no lesion swab samples were collected during the previous mission, saliva samples taken in flight were positive for EBV and VZV. Immunologic, endocrine, biochemical, and viral sequencing data from this long-duration ISS spaceflight suggest a viral association to the symptoms observed in this astronaut.

## 2. Materials and Methods

### 2.1. Study Subject

**Figure 1:**
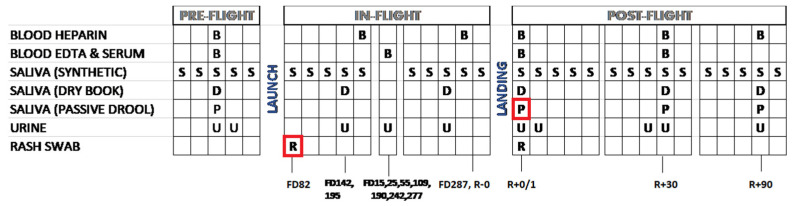
Schedule for collection of biological samples aboard ISS. Samples sent for sequencing are boxed in red. B—blood; S—liquid saliva; D—dry saliva; P—passive drool; U—urine; R—return after flight; FD—flight day. The subject was HSV-1 seropositive.

The subject is an astronaut who participated in several concurrent NASA experiments, which were approved by the Johnson Space Center Institutional Review Board. Written informed consent was provided prior to data acquisition. The astronaut provided verbal and written approval to publish these data. The NASA Lifetime Surveillance of Astronaut Health Project personnel reviewed and approved the sharing of data among the experiments and approved the publication of these data. The astronaut completed over 9 months onboard the ISS. Biological samples were collected before, during, and after the space mission, as described in Figure 1.

### 2.2. Saliva Sample Collection

Saliva was collected in three different ways (rolled, passive, and dry) to evaluate very specific outcome variables. Rolled saliva was collected for five consecutive days at each of the eight time points to evaluate viral load, alpha amylase, cortisol, and DHEA. Passive drool was collected to evaluate the viability of infectious virus therein. Dry saliva was collected to determine the cortisol and DHEA concentration changes occurring during the day from wake to sleep.

Saliva was collected for eight mission time points: two pre-flight (180 and 45 days before flight), three in-flight (early, FD120; mid, FD180; and late, FD240), and three post-flight (1, 30, and 90 days after return) using a synthetic salivette (SalivaBio Oral Swab (SOS), Salimetrics, State College, PA, USA) each time immediately after their sleep cycle, before eating and brushing their teeth [5]. These samples were stored frozen until processed for viral DNA analysis by real-time PCR. Samples collected in flight were stored frozen until returned to Earth by SpaceX vehicles and then transported to the Immunology Laboratory of Johnson Space Center for processing.

Diurnal dry saliva samples (5 per sampling day) were collected at awakening, wake +30 min, +6 h, +10 h, and then upon retiring, using a unique filter paper collection method. The subject wet the filter paper with saliva, which was then air-dried and stored at room temperature until return to Earth. All of a subject’s samples were assayed in batch on the same plate. Filters were processed for cortisol and DHEA measurements as previously described [14]. Intra and inter-assay coefficients of variation for cortisol and DHEA were less than 5% and 10%, respectively, using this procedure.

To culture live virus, fresh saliva was collected by passive drool method one day after landing and processed immediately as follows: the sample was centrifuged, and the cell pellet was re-suspended in one milliliter of DMEM (Dulbecco’s Modified Eagle Medium, Gibco, Invitrogen, Carlsbad, CA, USA) media. The cells were then plated atop human lung fibroblasts (HFL), then the culture vessel was centrifuged at 500 rpm for 5 min and then incubated at 37 °C with 5% humidified CO_2_. The cultures were observed daily for the formation of viral plaques, which were in turn confirmed by HSV-1-specific antibody staining.

### 2.3. DNA Extraction from Biological Samples

A single peripheral blood sample using an 8.5 mL heparin anti-coagulated tube and a 24 h urine pool were also collected, each mission time point (Figure 1). Only one blood sample was taken at each in-flight time point. DNA was extracted from all the frozen and fresh saliva samples, as well as blood samples using a QIAamp DNA Blood Mini Kit (Qiagen, Hilden, Germany). DNA was extracted from urine samples using QIAamp Viral RNA mini kit (Qiagen, Hilden, Germany). The viral loads (viral copies/ng DNA recovered) for EBV, HSV-1, VZV, and CMV were determined by real-time PCR using TaqMan 7900 [4,5]. The primers and probes used for EBV, VZV, and CMV have been published previously [5].

### 2.4. Blood Samples

To correlate the reactivation of latent herpesviruses with immune system dysregulation, an immune assessment was conducted in conjunction with saliva collection. A blood sample was collected twice during flight; each collection was near the time of hatch closure for a returning Soyuz spacecraft. The blood samples were returned in ambient conditions to support live cellular functional analyses.

### 2.5. Immunology and Biochemical Assays

Peripheral leukocyte distribution, plasma cytokine analysis, T-cell function, and mitogen-stimulated cytokine profiles were determined as previously described [1,10]. Additional blood samples (using serum and EDTA plasma separator tubes) were collected at seven distinct time points as part of a separate ‘Biochemical Profile’ study activity (FD15, 25, 55, 109, 190, 242, and 277). Samples were centrifuged and immediately frozen in a −80 °C freezer onboard ISS for return to Earth as previously described [15]. Once the samples returned to Earth, the serum and plasma were aliquoted and frozen until batch analysis. Assays included general chemistry, vitamin and mineral status, hormone, bone metabolism, and renal stone assessments as described [16,17].

### 2.6. Skin Swab

A skin lesion swab was collected on FD82 using an EnviroTrans™ swab rinse kit containing 5 mL 0.85% saline with a swab (Hardy Diagnostics, Santa Maria, CA, USA). The sample was stored frozen at −80 °C until processed in the laboratory as follows: The sample was homogenized, and the cell pellet was separated by centrifuging at 14,000 rpm for 15 min. The DNA extraction for PCR was performed on one part of the sample while the other part was plated onto HFL cells (ATCC CCL-153) for viral culture. Viral load was measured in triplicate, and the average was normalized by the DNA concentration. DNA concentration was determined using a Qubit 2.0 Fluorometer and Invitrogen™ Quant-iT™ Qubit™ dsDNA HS Assay Kits (Invitrogen, Carlsbad, CA, USA). HSV-1 primers and probe sequences used for qPCR were as follows: forward primer (TGG TAT TGC CCA ACA CTT TCC), reverse primer (GCG CCA GGC ACA CAC AT), and probe (CGT GTC GCG TGT GGT).

### 2.7. Clinical Specimen DNA and HSV Genome Quantitation

Isolated DNA samples were sent to Pennsylvania State University for genome quantification. Samples included DNA isolated from both the cell pellet and supernatant of each virus-positive sample: one in-flight rash swab (FD 82) and one post-flight (R + 0) passive saliva sample. Following acquisition of the clinical specimens, 20 μg of linear polyacrylamide was added to each sample to serve as an inert co-precipitant. Total DNA was quantified using a Qubit^®^ 2.0 fluorimeter with Qubit^®^ compatible High-Sensitivity assay reagents (Invitrogen #Q32854). Viral DNA was quantified using qPCR to detect the type-common region of HSV glycoprotein B (gB) gene U_L_27 as previously described [18,19]. This value was then used to infer the number of gB copies and, by inference, the total number of HSV copies in each sample.

### 2.8. Library Prep, Oligonucleotide Enrichment, and Illumina Deep Sequencing

Total sample DNA was sheared into ~800 base-pair (bp) fragments using a Covaris sonicator with settings as follows: 10% duty, power 60, 200 cycles/burst for 60 s at 4 °C. Sheared DNA was processed using the KAPA Hyperprep Kit (KAPA Biosystems #KR0961) compatible with Illumina^®^ platform reagents. Following overnight ligation with Illumina indices at 4 °C, a post-ligation cleanup was performed before library DNA was amplified by PCR (10–14 cycles). The oligonucleotide bait library was a custom Arbor Biosciences target enrichment platform designed and validated in house [20]. The bait library included custom biotinylated DNA fragments (MyBaits^®^, Arbor Biosciences, Ann Arbor, MI, USA) that bind specifically to the DNA of HSV-1 strain 17 (GenBank JN555585.1). Each sample library was hybridized for ~40 h at 65 °C with the oligonucleotide probes, after which the baits and attached captured viral DNA were isolated using streptavidin-bound magnetic beads. Following enrichment, a post-capture PCR step (14 cycles) was performed before sequencing each bar-coded library on an Illumina MiSeq to obtain 300 bp paired-end sequence reads using v.3 chemistry.

### 2.9. De Novo Viral Genome Assembly

Following paired-end Illumina sequencing, the sequence reads were passed through a series of quality control filters to remove Illumina primers and adapters [21,22]. A BLAST database consisting of all known HSV-1 and HSV-2 genomes was constructed, and sequence reads with an e-value less than 10^2^ were used to build consensus genomes. Consensus HSV-1 genomes were assembled de novo using a previously published viral genome assembly pipeline (VirGA) [23]. Briefly, eight SSAKE de novo assemblies were generated and combined into a draft genome for each sample using Celera and GapFiller [24,25,26]. The consensus HSV-1 genomes were annotated based on the HSV-1 reference genome (strain 17; GenBank ID JN555585) using sequence homology. The supernatant- and pellet-derived viral DNA was sequenced separately for each sample (in-flight swab and post-flight saliva), and the FASTQ data files were combined after Illumina sequencing. GenBank IDs and sequencing statistics for the two clinical genomes generated in this work are listed in Table 1.

### 2.10. Consensus Genome Comparison and Phylogenetic Analysis

Consensus genome comparisons between the two HSV-1 genomes were performed using trimmed versions of viral genomes (lacking the terminal repeats) so as to avoid over-representation of the internal and terminal repeats [27]. MAFFT was used to construct pairwise global nucleotide alignments between whole genome sequences [28]. ClustalW2 was used for pairwise amino acid alignments between open reading frames [29]. Custom Python scripts were used to calculate protein-coding differences and DNA variation between samples. The phylogenetic network was constructed with SplitsTree 4.14.5, using the uncorrected P distance and excluding all gaps [30]. See Supplementary Table S1 for GenBank IDs and references for HSV-1 isolates used to construct the phylogenetic network.

### 2.11. Minor Variant Detection and Validation

Each consensus genome was analyzed for the presence of minor variant (MV) loci, which are defined as alternative alleles that exist at a low frequency (≤50%) in the viral population. VarScan v2.2.11 was used to detect MVs in each population of viral genomes [31]. Multiple parameters were applied to differentiate true MVs from potential sequencing artifacts [32]: minimum variant allele frequency ≥ 2% (0.02); base call quality ≥ 20; read depth at the position ≥ 100; independent reads supporting minor allele ≥ 5. Polymorphisms with directional strand bias ≥ 90% were excluded. SnpEff and SnpSift were used to annotate the MVs and identify their distribution and potential impact [33,34]. All MVs were visually inspected using the Integrative Genomics Viewer (IGV) v2.3.97 to verify raw sequence read support for the VarScan output.

### 2.12. Statistical Analyses

Pearson correlation constants were determined for relationships between angiotensin II and cytokines and markers of oxidative damage. One-way ANOVA with Sidak’s multiple comparison tests was used to determine the significance of the averages of the 5 saliva samples collected at each time point compared to baseline (L-180) for DHEA, cortisol, and alpha amylase. Additionally, one-way ANOVA with Sidak’s multiple comparison tests was used to calculate the significance of the averages between each phase of the mission (pre-, during-, and post-mission) for DHEA, cortisol, and alpha amylase.

## 3. Results

### 3.1. Stress Hormones and Biochemistry

**Figure 2:**
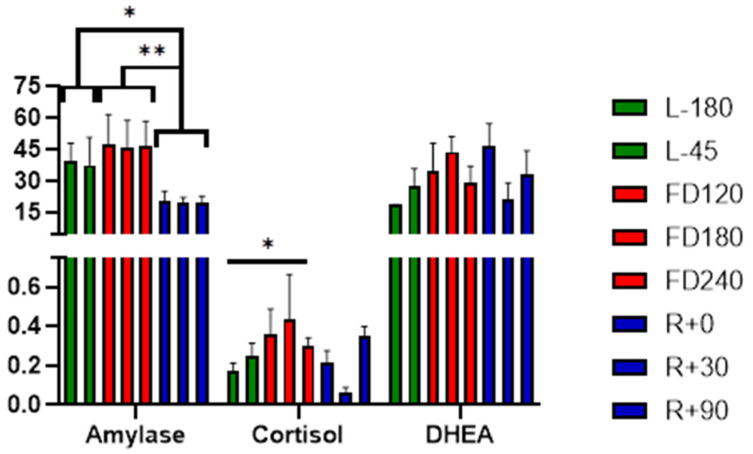
Mean distribution of salivary alpha amylase, cortisol, and DHEA before (green), during (red), and after (blue) the mission. Pre-mission samples are indicated by “L-“ referencing the number of days before launch. In-flight mission samples are indicated as FD, flight day. Post-mission samples are similarly indicated by “R+” for the days post-return. Statistical significance is indicated by * *p* < 0.05 and ** *p* < 0.01.

The mean of salivary amylase, cortisol, and DHEA measured before, during, and after the flight are given in Figure 2. Salivary cortisol, a marker of activation of the HPA axis, revealed higher concentrations during flight, FD120, FD180, and FD240 as compared to before and after flight (Figure 2). A statistically significant increase was observed at FD240 (0.301 ± 0.042 μg/DL) when compared to baseline at L-180 (0.172 ± 0.040 μg/DL; *p* = 0.023). Circadian rhythm of cortisol done based upon five samples collected during the day (explained in Methods section for dry saliva) did not show any significant changes at any of the time points measured during the mission. Salivary DHEA also showed an increasing trend during early and mid-flight but did not show any significant changes between the phases. No significant increase in alpha amylase, a biomarker for assessing acute psychological stress in humans due to activation of the sympathetic nervous system, was observed during flight as compared to pre-flight at any of the time points tested. However, when combining the pre-flight, during-flight, and post-flight sessions, post-flight alpha amylase levels (mean ± SEM 20.1 ± 1.89 U/mL) were significantly decreased compared to both pre-flight (mean ± SEM 38.4 ± 7.48 U/mL; *p* = 0.046) and during flight (mean ± SEM 46.6 ± 6.95 U/mL; *p* = 0.004).

Several analytes increased during flight are consistent with an inflammatory response and/or oxidative stress, including ferritin, and serum and urinary cortisol (Supplementary Table S2). Folate status was at the low end of the clinical normal range during the mission (in-flight concentrations ranged from 21 early during flight to 13 nmol/L by the end of the mission, normal range 21–64 nmol/L). Vitamin B6 (pyridoxal 5-phosphate) decreased over the course of the mission from 48 nmol/L before flight to 16 on landing day (normal range 11.3–302 nmol/L). Angiotensin II increased as much as 80% early during flight, and this mirrored many of the inflammatory cytokines (see raw data in Supplementary Table S2—Biochemical profile). Angiotensin II was correlated with TNFα (Pearson *r* = 0.60, *p* = 0.04) and markers of oxidative damage (oxidized LDL, Pearson *r* = 0.72, *p* = 0.07; 8-hydroxy 2′-deoxyguanosine, Pearson *r* = 0.84, *p* = 0.02).

### 3.2. Immune Status

**Figure 3:**
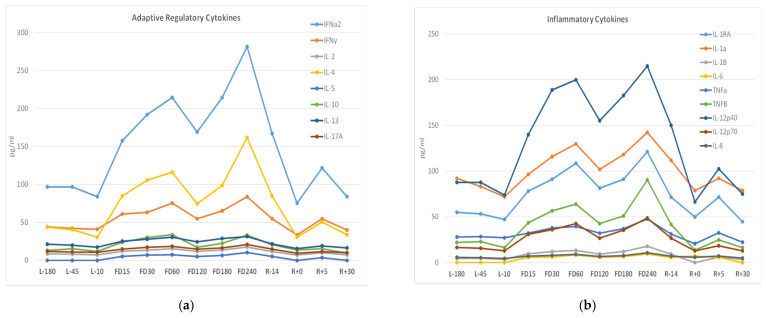
Adaptive regulatory (**a**) and inflammatory (**b**) plasma cytokines concentrations (pg/mL) measured via multiplex array before, during, and after the spaceflight. Pre-flight samples are indicated by “L-“ referencing the number of days before launch. In-flight (flight day) mission samples are indicated by “FD” followed by the number of days post launch. Post-flight samples are similarly indicated by “R+” for the days post-return.

The astronaut subject displayed a fairly typical pattern of immune changes previously characterized as being associated with spaceflight [4,10,11]. There was little alteration in the distribution of peripheral leukocytes and a mild reduction in T-cell function as determined by the induction of cellular activation antigens following mitogenic stimulation via Staphylococcal enterotoxins or antibodies to CD3 and CD28 as previously described [1,10] (data not shown). Mitogen-stimulated cytokine profiles did not display the characteristic reduction in cytokine induction observed in previous astronaut studies (data not shown). An assessment of plasma cytokine concentrations revealed an elevation during mission in several cytokines, which is not atypical for astronauts during spaceflight (Figure 3). Plasma cytokine concentrations across three pre-flight samples were very consistent. Several inflammatory cytokines, including IL-1, IL-1ra, and IL-12, as well as cytokines associated with adaptive immune responses including IFNa, IFNγ, and IL-4, were elevated throughout the mission, Figure 3.

### 3.3. Viral PCR Analysis

The levels of three herpesviruses, HSV-1, EBV, and VZV, were quantified in 40 saliva samples collected at different times before, during, and after the spaceflight by real-time PCR. No EBV or VZV was detected by real-time PCR in any of the samples collected before, during, and after spaceflight. None of the eight 24 h urine samples collected during this mission was positive for CMV. Even though no HSV-1 was detected before the flight, the saliva sample collected on the 82nd day of the spaceflight was positive for HSV-1 (4.8 copies/ng DNA). The rash swab collected from a skin lesion from the neck region on the same flight day was also positive for HSV-1 (5.3 × 10^4^ copies/ng DNA). Saliva collected at landing (R + 0) showed 1.67 × 10^7^ copies/ng of total DNA. The post-flight passive saliva yielded infectious HSV-1, as observed by culturing on HFL cells. Post-recovery saliva and skin swab samples collected 30 and 90 days after spaceflight did not test positive for viral DNA, which coincided with negative virus recovery by culture as well as a lack of symptoms.

### 3.4. Viral Genome Sequencing and Comparison

**Figure 4:**
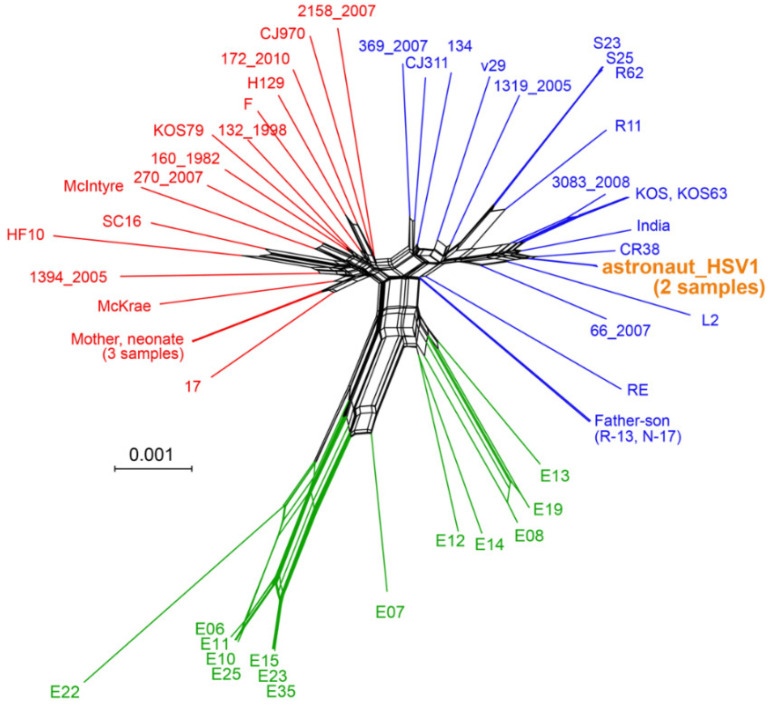
Network graph demonstrating the relatedness of astronaut and other HSV-1 genomes. The two astronaut-derived HSV-1 genomes (orange) clustered nearest to HSV-1 genomes from China (CR-38) and Russia (L2). This graph-based SplitsTree network was generated from an alignment of the astronaut-derived HSV-1 genomes with 51 HSV-1 genomes that encompass the global genetic diversity of this virus. The Φ statistical test in SplitsTree4 found statistically significant evidence for recombination (*p* = 0.0), as expected based on prior analyses of HSV-1 phylogenies. The overall geographical origins of the prior HSV-1 genomes are European and North American (red); European, North American, Asian (blue), and African (green). The scale bar represents 0.1% nucleotide divergence. Supplementary Table S1 includes the names, accessions, and geographic origins of each strain.

In order to gain insight on whether the in-flight rash associated with HSV-1 shedding was identical to the viral DNA detected in saliva at landing post-flight (R + 0), we subjected DNA isolated from these samples to library preparation and oligonucleotide-bait-based enrichment for HSV-1 DNA. We then used this virus-enriched library for high-throughput deep viral genome sequencing (see Table 1 for DNA isolation yield, enrichment, and genome metrics). The resulting sequence data were de novo assembled into full-length HSV-1 genomes using our previously published methods [23]. Comparison of these genomes to each other revealed them to be 99.9% identical at the consensus genome level, i.e., the most frequent base detected at each position in the viral genome. We compared the astronaut’s two viral consensus genomes to a set of 51 previously described and sequenced HSV-1 genomes (Supplementary Table S1), using a network graph (Figure 4). This analysis revealed the astronaut’s HSV-1 genomes to be most similar to HSV-1 genomes previously collected in China (CR38, HM585508 [27]) and Russia (L2, KT780616 [35]) (Figure 4). While the Russian sample is extant, the CR-38 sample was collected in the 1980s [27]. Relative to this collection of known viral genomes, there were only four novel and unique amino acid (AA) coding variations in these astronaut HSV-1s (Table 2).

### 3.5. Minor Variants Indicate Higher Viral Genetic Diversity for In-Flight Sample

**Figure 5:**
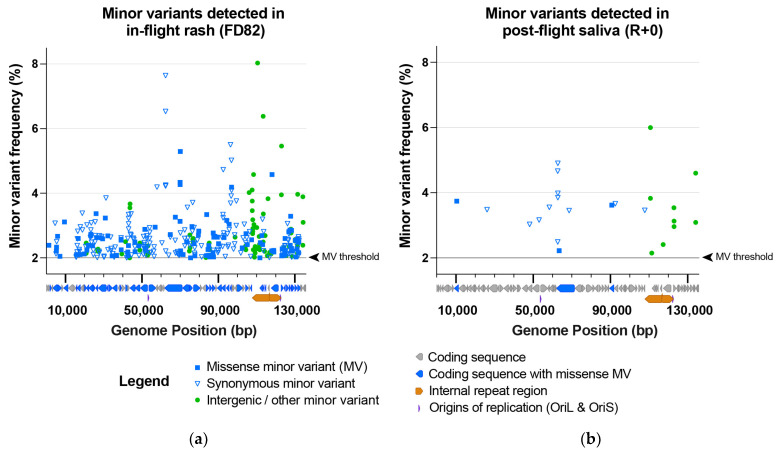
The in-flight rash (FD82) HSV-1 sample contained far more minor variants in the viral genome population than the post-flight saliva (R + 0) HSV-1 sample. This included (**a**) 366 MVs in the in-flight rash vs. (**b**) 24 in the post-flight saliva HSV-1 samples. Minor variants (MVs) are plotted based on their location within the HSV-1 genome (x-axis) and the frequency of the detected MV allele (y-axis) at each position. Minor variants are color-coded according to their classification as missense (genic), synonymous (genic), or intergenic/other. A black line and arrowhead (right of each plot) indicate the MV detection threshold of 2% (0.02). A diagram of HSV-1 coding sequences is located below each x-axis to highlight the coding sequences that contain missense minor variants. Supplementary Table S3 includes the precise location, coverage depth (i.e., forward and reverse reads supporting the major vs. the minor allele), and a list of specific MV impacts within each gene.

Since prior studies of direct-from-patient viral shedding have revealed within-host variation in the viral genome population, we next investigated whether the deep sequencing coverage of the astronaut’s HSV-1 genomes would reveal any variation within the virus population of each sample (average coverage > 11,000-fold; see Table 2). We used a conservative limit of detection to identify only those minor variants present in ≥2% of the population (see Methods for full criteria). This analysis identified minor variants (MV) at 366 sites in the in-flight rash HSV-1 population, but only 24 sites in the post-flight saliva HSV-1 population (Figure 5). These minor variants were distributed throughout the viral genome (Figure 5). With the exception of four synonymous MVs that occur in a tandem repeat (the “PQ” repeats) in the gene UL36, encoding the tegument protein VP1/2, there were no MVs in common between these two samples.

## 4. Discussion

Herpes simplex virus-1 (HSV-1) is a highly prevalent and communicable neurotropic alpha herpesvirus that establishes latency in cranial nerve ganglia. This virus persists lifelong as a latent virus and is commonly referred to as oral herpes. Reactivation of HSV-1 can be asymptomatic or can lead to lesions or skin rashes at sites corresponding to the infected innervating ganglion. The most typical presentation is characterized by an oral “cold sore” lesion. Reactivation of latent herpesviruses has been reported in previous spaceflight studies, and it largely is an asymptomatic phenomenon. However, in some astronauts, herpesvirus reactivation may potentially lead to clinical diseases such as zoster and dermatitis. Here, we report for the first time, detection of HSV-1 both in saliva and in skin lesions of an astronaut during long-duration spaceflight. The astronaut evaluated had persistent dermatitis during flight, with atypical presentation but also cold sores, which were treated with a combination of antihistamines, topical and oral steroids, and Valtrex. No HSV-1 DNA was detected in the astronaut’s samples collected before flight at 180 and 45 days before launch. Samples collected during spaceflight were found to have a viral HSV-1 load by real-time PCR, with 4.8 copies/ng in a saliva sample and 5.3 × 10^4^ copies/ng in a lesion swab sample. The post-landing saliva samples yielded infectious HSV-1 with extremely high copy numbers (1.67 × 10^7^ copy/ng DNA). This was confirmed by specific antibody staining as well as by PCR. HSV-1 genomes recovered from the in-flight skin lesion and post-orbit saliva were genotyped and found to have 99.9% homology.

It is generally established that reductions in immunocompetence, including T-cell function, can result in loss of “control” of latent herpesviruses and thus allow their reactivation. Such immune suppression is also typically associated terrestrially with either stress or aging. However, astronauts who are typically very fit individuals in an extreme environment also experience latent herpesvirus reactivation associated with immune suppression. This phenomenon likely results from a synergy of stress, microgravity, radiation, and/or prolonged isolation. As observed in space missions with previous astronauts, this astronaut did show increases in several salivary markers of stress (Figure 2) during flight compared to pre- and post-flight, confirming physiological stress changes within this extreme situation. Surprisingly, the current subject’s stress levels did not manifest the broad suppression in T-cell function observed in previous studies by the reduction in mitogen-stimulated cytokine production. However, a more atypical immune dysregulation did occur, as evidenced by the elevation in many plasma cytokines spanning the entire mission duration (Figure 3). We speculate that this astronaut subject retained immune cellular functions and stress hormone stability more so than the astronaut norm, yet the dysregulation and stress imbalance was still sufficient to manifest HSV-1 reactivation and shedding. Alternatively, the reactivation may be associated with loss of virus control by an alternate and unmonitored cellular mechanism such as angiotensin II discussed below.

Our comparison of in-flight vs. post-flight HSV-1 genomes revealed that these viral genomes were nearly identical to one another (99.94%) at the consensus level. However, the in-flight HSV-1 genome had strikingly more minor variants (366) than the post-flight sample (24; see Figure 5). This numeric difference alone suggests that there are distinct environmental or clinical impacts on these viral populations during spaceflight. From an environmental perspective, the in-flight sample contains viral genomes that replicated in the presence of ionizing radiation, which may have impacted the rate of double-strand DNA breaks, recombination, and DNA repair, as well as impacts on the host DNA damage response [36,37,38,39]. However, we cannot untangle that possibility from other clinical distinctions between these samples, such as their different status of viral reactivation. For instance, an active lesion and ongoing rash during space flight may entail different viral and host responses than asymptomatic viral shedding in saliva after landing. There is also a potential difference in immune surveillance, such as inflammatory or immune cell-killing states, at the site of a skin lesion versus at salivary sites of asymptomatic viral shedding. Distinguishing between these possibilities will necessitate analysis of further in-orbit shedding samples, both asymptomatic and symptomatic, as well as additional comparisons of pre- and post-flight samples from future space travel.

Nutritional status decreased during flight for some nutrients, particularly folate, vitamin B6, niacin, and vitamin C (see Supplementary Table S2). Total and LDL cholesterol increased as well as triglycerides. The increase in angiotensin II could have contributed to an inflammatory process that contributes to viral reactivation. Angiotensin II exerts an inflammatory effect on leukocytes and endothelial cells, contributing to reactive oxygen species formation, adhesion molecule, and chemokine release [40]. Angiotensin II also modulates T-cell activation through autocrine actions from endogenous production. T cells can produce angiotensin II, which stimulates the production of superoxide, which promotes T cells to produce TNFα [41]. In the current subject, angiotensin II concentration was positively correlated with both TNFα and markers of oxidative damage (oxLDL and 8-hydroxy 2′-deoxyguanosine). Supportive of this, there is a higher prevalence of HSV-1 activation in patients with chronic kidney disease as an inflammatory stimulus where angiotensin II is high [42].

Regardless of the cause, this subject showed symptomatic in-flight HSV-1 viral reactivation and asymptomatic, continued shedding of infectious HSV-1 virus upon landing, which potentially could have transferred and caused clinical disease in crew contacts, including uninfected or immunocompromised individuals, as well as newborn infants. Specifically, infants exposed to HSV-1 are at increased risk for developing severe and life-threatening complications such as fatal organ damage (liver, lungs, and heart), as well as viral meningitis and sores on the face and eyes. Thus, it is essential to develop spaceflight countermeasures to prevent HSV-1 reactivations and ensure the health of the crew, as well as the health of their contacts upon return. One countermeasure that has proven efficacious is viral-specific therapeutic vaccines such as Zostavax against VZV reactivation. Our previous work on the reactivation of VZV in astronauts led to the implementation of Zostavax as a prophylactic countermeasure to herpes zoster. Since its implementation in 2013, very little VZV reactivation has been observed in crew samples. Interestingly, with the reduction in VZV reactivation, we have observed an increasing incidence of subclinical HSV-1 reactivation in crew samples. Historically, HSV-1 reactivation rates have been very low, but they now seem to be on the rise, suggesting a potential inverse correlational relationship with VZV reactivation. That may be true for the subject of this case study. In a previous long-duration mission, this astronaut had dermatitis and VZV and EBV shedding, although EBV would be an unlikely source of skin lesions. In the current study, this astronaut had dermatitis that has been directly linked to HSV-1 reactivation, and there was no evidence, in any of the biological samples, for VZV reactivation. No VZV vaccination was given between the missions. Unfortunately, at this time, no HSV-1 vaccine is available, but several therapeutic live-attenuated and subunit vaccines are in clinical or pre-clinical trials [43]. Another potential countermeasure against both HSV-1 and VZV, especially for individuals for whom vaccines are either ineffective or contraindicated, could be prophylactic administration of an antiviral drug (valacyclovir or Valtrex). Interestingly, the astronaut from this study was treated with Valtrex (2000 mg PO q12h for 2 days) during this mission (flight days 53 and 168) upon outbreak of cold sores, but it did not seem to be efficacious. Perhaps prophylactic pre-dosing and/or using a higher dose during outbreak could have reduced or eliminated the viral and symptom burden. To that end, we are currently studying the effectiveness of this prophylactic antiviral countermeasure to potentially prevent VZV and HSV-1 (as well as EBV) shedding in Antarctic expeditioners, who have similar patterns of viral DNA shedding in saliva as astronauts. These and other countermeasures (i.e., specific resistive and aerobic exercise, nutritional supplementation, and stress relieving exercise) must continually be evaluated and updated to ensure the health and safety of our astronauts in space and their contacts upon return.

## 5. Conclusions

These data confirm that in some cases, the viral reactivation, stress imbalance, and immune dysregulation experienced by astronauts during spaceflight is not entirely a subclinical phenomenon and may indeed be associated with clinical processes requiring treatment. For this case, dermatological lesions present during space flight and immediately after returning to Earth coincided with elevated stress markers, circulating inflammatory cytokines, and HSV-1 DNA shedding in saliva and lesion swab. HSV-1 genomes recovered from in-flight skin lesions and post-orbit saliva were deep-sequenced and found to be nearly identical at the consensus genome level. However, the in-flight rash sample contained a far more diverse population of viral genomes than the post-orbit saliva sample. The reasons for this are not known but may include differences in immune activation state, exposure to ionizing radiation in space, or lesion vs. asymptomatic shedding. Astronaut saliva contains increasingly significant viral DNA, which can be infectious during and after spaceflight. For that reason, and in response to the data from the current astronaut subject indicating that the persistent skin rash may have a viral etiology, we recommend prophylactic (vaccine and/or antiviral) treatment, where available, to the astronauts before they go into space as a countermeasure. Further, a broader package of countermeasures to reduce viral reactivation and restore immune function should be considered for the upcoming “Artemis” deep space missions.

## Figures and Tables

**Table 1:** Sequencing statistics and GenBank IDs for two HSV-1 genomes from NASA astronaut.

Virus	GenBank ID	Input DNA ^^^ HSV Copy #	Enriched Library ^^^ HSV Copy #	Total # Reads *	% HSV	# Reads Used for Assembly *	Average Coverage
In-flight rash (FD82)	ON152715	1.7 × 10^3^	5.7 × 10^5^	7.8 million	80%	6.2 million	11,166X
Post-flight (R + 0 passive) saliva	ON152716	2.0 × 10^7^	2.5 × 10^7^	8.6 million	79%	6.8 million	11,828X

**^^^** Values determined by qPCR on each sample after DNA isolation (see Methods for details). * All read counts refer to the number of forward reads only. “Total # reads” includes all forward reads, including unpaired and host-aligning reads. “# reads used for assembly” includes quality-trimmed, properly paired, HSV-specific forward reads.

**Table 2:** Unique amino acid variants found in astronaut’s HSV-1 genomes.

Gene	Gene Product	AA Alignment Position	Astronaut HSV-1 AA	Wildtype AA
UL8	DNA helicase/primase	595	T	A
RL1	ICP34.5, neurovirulence factor	155	T	A
US2	Unknown function	166	Q	P
US5	Glycoprotein J	52	A	V

## Data Availability

The data presented in this study are available on request from the corresponding author and approval through NASA. The data are not publicly available, with the exception of the two viral genomes deposited in GenBank.

## Supplementary Materials

The following supporting information can be downloaded at: https://www.mdpi.com/article/10.3390/v14040789/s1, Table S1: GenBank accession numbers and references; Table S2: Biochemical profile; Table S3: Minor variant details.

## Legal

Trade names and trademarks are used in this report for identification only. Their usage does not constitute an official endorsement, either expressed or implied, by the National Aeronautics and Space Administration.
