# Supplementary material for: Dermatitis during Spaceflight Associated with HSV-1 Reactivation
Source: Viruses. 2022 Apr 11;14(4):789. doi: 10.3390/v14040789 (PMC9028032; doi:10.3390/v14040789)
Supplement: Supplementary file 1 [file viruses-14-00789-s001.zip › Supplemental_Table_S1_NASA_HSV1_v2.pdf]

Supplemental Table S1: GenBank accession numbers and references for previously sequenced isolates used in comparative genomics and phylogenetic analyses.

Virus	Strain Origin	GenBank Accession	Refs.*
17	Glasgow, UK	JN555585 NC_001806	(1, 2)
F	Chicago, IL	GU734771	(3, 4)
H129	San Francisco, CA	GU734772	(3, 5)
KOS	Houston, TX	JQ673480, JQ780693	(6, 7)
McKrae	Gainesville, FL	JQ730035, JX142173	(8–10)
HF10	New York, NY	DQ889502	(11)
KOS63	Houston, TX	KT425110	(12)
KOS79	Houston, TX	KT425109	(12)
India	Pune, India	KJ847330	(13)
L2	Moscow, Russia	KT780616	(14)
SC16	Madrid, Spain	KX946970	(15)
MacIntyre	Berkeley, CA	KM222720	(16)
CJ970	Madison, WI	JN420341.1	(17)
CJ311	Madison, WI	JN420338.1	(17)
134	Madison, WI	JN4000093.1	(18)
RE	New Orleans, LA	KF498959	N/A
160/1982	Erfurt, Germany	LT594192	(19)
132/1998	Gelsenkirchen, Germany	LT594457	(19)
1394/2005	Germany	LT594111	(19)
1319/2005	Germany	LT594108	(19)
66/2007	Jena, Germany	LT594110	(19)
369/2007	Jena, Germany	LT594112	(19)
3083/2008	Jena, Germany	LT594107	(19)
270/2007	Manebach, Germany	LT594109	(19)
2158/2007	Jena, Germany	LT594106	(19)
172/2010	Jena, Germany	LT594105	(19)
CR38	Shenyang, China	HM585508	(20)
E07	Nairobi, Kenya	HM585497	(20)
E06	Nairobi, Kenya	HM585496	(20)
E08	Nairobi, Kenya	HM585498	(20)
E10	Nairobi, Kenya	HM585499	(20)
E11	Nairobi, Kenya	HM585500	(20)
E12	Nairobi, Kenya	HM585501	(20)
E13	Nairobi, Kenya	HM585502	(20)
E14	Nairobi, Kenya	HM585510	(20)
E15	Nairobi, Kenya	HM585503	(20)
E19	Nairobi, Kenya	HM585511	(20)
E22	Nairobi, Kenya	HM585504	(20)
E23	Nairobi, Kenya	HM585505	(20)
E25	Nairobi, Kenya	HM585506	(20)
E35	Nairobi, Kenya	HM585507	(20)
R11	Seoul, South Korea	HM585514	(20)
R62	Seoul, South Korea	HM585515	(20)
S23	Sapporo, Japan	HM585512	(20)
S25	Sapporo, Japan	HM585513	(20)
N-7	Cincinnati, OH	KY922719	(21)
R-13	Cincinnati, OH	KY922718	(21)
v.29	Seattle, WA	MH102298	(22)
mother_blood	USA	MK952185	(23)
neonate_skin	USA	MK952183	(23)
neonate_blood	USA	MK952184	(23)

References

1. McGeoch, D.J.; Dalrymple, M.A.; Davison, A.J.; Dolan, A.; Frame, M.C.; McNab, D.; Perry, L.J.; Scott, J.E.; Taylor, P. The complete DNA sequence of the long unique region in the genome of herpes simplex virus type 1. *J. Gen. Virol.* **1988**, *69*, 1531–1574.
2. McGeoch, D.J.; Dolan, A.; Donald, S.; Rixon, F.J. Sequence determination and genetic content of the short unique region in the genome of herpes simplex virus type 1. *J. Mol. Biol.* **1985**, *181*, 1–13.
3. Szpara, M.L.; Parsons, L.; Enquist, L.W. Sequence Variability in Clinical and Laboratory Isolates of Herpes Simplex Virus 1 Reveals New Mutations. *J. Virol.* **2010**, *84*, 5303–5313. https://doi.org/10.1128/jvi.00312-10.
4. Ejercito, P.M.; Kieff, E.D.; Roizman, B. Characterization of herpes simplex virus strains differing in their effects on social behaviour of infected cells. *J. Gen. Virol.* **1968**, *2*, 357–364
5. Dix, R.D.; McKendall, R.R.; Baringer, J.R. Comparative neurovirulence of herpes simplex virus type 1 strains after peripheral or intracerebral inoculation of BALB/c mice. *Infect. Immun.* **1983**, *40*, 103–112.
6. Macdonald, S.J.; Mostafa, H.H.; Morrison, L.A.; Davido, D.J. Genome sequence of herpes simplex virus 1 strain KOS. *J. Virol.* **2012**, *86*, 6371–6372.
7. Smith, K.O. Relationship Between the Envelope and the Infectivity of Herpes Simplex Virus. *Exp. Biol. Med.* **1964**, *115*, 814–816.
8. Watson, G.; Xu, W.; Reed, A.; Babra, B.; Putman, T.; Wick, E.; Wechsler, S.L.; Rohrmann, G.F.; Jin, L. Sequence and com-parative analysis of the genome of HSV-1 strain McKrae. *Virology* **2012**, *433*, 528–37.
9. Macdonald, S.J.; Mostafa, H.H.; Morrison, L.A.; Davido, D.J. Genome sequence of herpes simplex virus 1 strain McKrae. *J. Virol.* **2012**, *86*, 9540–9541.
10. Williams, L.E.; Nesburn, A.B.; Kaufman, H.E. Experimental induction of disciform keratitis. *Arch. Ophthalmol.* **1965**, *73*, 112–114.
11. Ushijima, Y.; Luo, C.; Goshima, F.; Yamauchi, Y.; Kimura, H.; Nishiyama, Y. Determination and analysis of the DNA se-quence of highly attenuated herpes simplex virus type 1 mutant HF10, a potential oncolytic virus. *Microbes Infect.* **2007**, *9*, 142–149.
12. Bowen, C.D.; Renner, D.W.; Shreve, J.T.; Tafuri, Y.; Payne, K.M.; Dix, R.D.; Kinchington, P.R.; Gatherer, D.; Szpara, M.L. Viral forensic genomics reveals the relatedness of classic herpes simplex virus strains KOS, KOS63, and KOS79. *Virology* **2016**, *492*, 179–186.
13. Bondrem, V.P.; Sankararaman, V.; Andhare, V.; Tupekar, M.; Sapkal, G.N. Genetic characterization of human herpesvirus type 1: Full-length genome sequence of strain obtained from an encephalitis case from India. Indian. *J. Med. Res.* **2016**, *144*, 750–760.
14. Skoblov, M.Yu.; Lavrov, A.V.; Bragin, A.G.; Zubtsov, D.A.; Andronova, V.L.; Galegov, G.A.; Skoblov, Yu.S. The genome nucleotide sequence of herpes simplex virus 1 strain L2. *Russ J. Bioorganic Chem.* **2017**, *43*, 140–142.
15. Rastrojo, A.; López-Muñoz, A.D.; Alcamí, A. Genome Sequence of Herpes Simplex Virus 1 Strain SC16. *Genome Announc.* **2017**, *5*, e01392-16.

16. Szpara, M.L.; Tafuri, Y.R.; Parsons, L.; Shreve, J.T.; Engel, E.A.; Enquist, L.W. Genome sequence of the antero-grade-spread-defective herpes simplex virus 1 strain MacIntyre. *Genome Announc.* **2014**, *2*, e01161-14.
17. Kolb, A.W.; Adams, M.; Cabot, E.L.; Craven, M.; Brandt, C.R. Multiplex Sequencing of Seven Ocular Herpes Simplex Virus Type-1 Genomes: Phylogeny, Sequence Variability, and SNP Distribution. Investig. *Opthalmology Vis. Sci.* **2011**, *52*, 9061.
18. Kolb, A.W.; Lee, K.; Larsen, I.; Craven, M.; Brandt, C.R. Quantitative Trait Locus Based Virulence Determinant Mapping of the HSV-1 Genome in Murine Ocular Infection: Genes Involved in Viral Regulatory and Innate Immune Networks Con-tribute to Virulence. *PLoS Pathog.* **2016**, *12*, e1005499.
19. Pfaff, F.; Growth, M.; Sauerbrei, A.; Zell, R. Genotyping of herpes simplex virus type 1 (HSV-1) by whole genome se-quencing. *J. Gen. Virol.* **2016**, *7*, 2732–2741. https://doi.org/10.1099/jgv.0.000589.
20. Szpara, M.L.; Gatherer, D.; Ochoa, A.; Greenbaum, B.; Dolan, A.; Bowden, R.J.; Enquist, L.W.; Legendre, M.; Davison, A.J. Evolution and diversity in human herpes simplex virus genomes. *J. Virol.* **2014**, *88*, 1209–1227.
21. Pandey, U.; Renner, D.W.; Thompson, R.L.; Szpara, M.L.; Sawtell, N.M. Inferred father-toson transmission of herpes simplex virus results in near-perfect preservation of viral genome identity and in vivo phenotypes. *Sci. Rep.* **2017**, *7*, 13666.
22. Shipley, M.M.; Renner, D.W.; Ott, M.; Bloom, D.C.; Koelle, D.M.; Johnston, C.; Szpara, M.L. Genome-wide surveillance of genital herpes simplex virus type 1 from multiple anatomic sites over time. *J. Infect. Dis.* **2018**, *218*, 595–605.
23. Shipley, M.M.; Renner, D.W.; Pandey, U.; Ford, B.; Bloom, D.C.; Grose, C.; Szpara, M.L. Personalized viral genomic inves-tigation of herpes simplex virus 1 perinatal viremic transmission with dual fatality. *Mol. Case Stud.* **2019**, *5*, a004382.
